# Antigen-Specific IgG Subclasses in Primary and Malignancy-Associated Membranous Nephropathy

**DOI:** 10.3389/fimmu.2018.03035

**Published:** 2018-12-20

**Authors:** Franziska von Haxthausen, Linda Reinhard, Hans O. Pinnschmidt, Michael Rink, Armin Soave, Elion Hoxha, Rolf A. K. Stahl

**Affiliations:** ^1^III. Medizinische Klinik und Poliklinik, Universitätsklinikum Hamburg-Eppendorf, Hamburg, Germany; ^2^Institut für Medizinische Biometrie & Epidemiologie, Universitätsklinikum Hamburg-Eppendorf, Hamburg, Germany; ^3^Klinik und Poliklinik für Urologie, Universitätsklinikum Hamburg-Eppendorf, Hamburg, Germany

**Keywords:** membranous nephropathy, IgG subclasses, malignancy, PLA_2_R1, THSD7A, prognosis

## Abstract

Membranous nephropathy (MN) is an autoimmune disease caused by binding of circulating antibodies to podocyte antigens in the kidney. For decades and still today primary MN has been considered to have an unspecified IgG4-driven autoimmune genesis, while secondary MN has been associated with other diseases, most notably cancer, and not linked to IgG4. Immunologic mechanisms of primary and malignancy-associated MN are assumed to be different, however, this has never been systematically evaluated. The identification of Phospholipase A_2_ Receptor 1 (PLA_2_R1) and Thrombospondin Type-1 Domain-Containing 7A (THSD7A) as target antigens in MN allows a pathogenesis-driven differential diagnosis. Recent data showing a molecular link between increased THSD7A-expression in tumors and THSD7A-antibody positive MN suggest a similar pathogenesis of malignancy-associated and primary MN. In order to better define the underlying immunologic processes, we systematically analyzed circulating antigen-specific IgG subclasses in the serum of 76 patients with PLA_2_R1-associated MN and 41 patients with THSD7A-associated MN in relationship to concurrent malignancy and disease outcome. Twenty-three patients in the study had malignancy-associated MN. We analyzed antigen-specific IgG subclasses in the serum of all patients at baseline and in 55 patients during follow-up by Western blot applying antigens derived from human kidney and lung. At baseline all 117 patients were positive for IgG4-antibodies against either PLA_2_R1 or THSD7A, while IgG3, IgG1, and IgG2-antibodies were found in 87, 72, and 26% of patients, respectively. There were no differences in the IgG subclass distribution between patients with primary vs. cancer-associated MN and no association with disease outcome. Moreover, levels of antigen-specific IgG4-antibodies were not different between primary and malignancy-associated MN and levels of all IgG subclasses did not differ between these groups. Both podocytes and lung bronchioles showed expression of both PLA_2_R1 and THSD7A when analyzed by immunofluorescence and Western blot. Every antigen-specific IgG subclass showed identical binding in both organs and autoantibodies bound the respective antigen only under non-reducing conditions. We conclude that antigen-specific IgG subclasses do not differentiate primary from malignancy-associated MN or predict disease prognosis. These data support the view that one common pathway may lead to primary and cancer-associated MN induced by PLA_2_R1- or THSD7A-antibodies.

## Introduction

Primary membranous nephropathy (MN) is an autoimmune disease caused by circulating antibodies against at least two known membrane proteins, Phospholipase A_2_ Receptor 1 (PLA_2_R1) and Thrombospondin Type-l Domain-Containing 7A (THSD7A), which are expressed on human podocytes (1, 2). The typical clinical presentation of disease is a nephrotic syndrome with proteinuria of more than 3.5 g/24 h. The clinical course of MN is unpredictable, ranging from spontaneous remission of proteinuria with excellent long-term outcome in one-third of patients to persistent nephrotic syndrome and end-stage renal disease in 20–40% of patients (3, 4). A decrease of autoantibody levels is associated with treatment response in patients with MN and usually precedes remission of proteinuria by months (5). The secondary form of MN is associated with other autoimmune, infectious or malignant diseases. Since definite proof for the etiologic causality relationship between these diseases and MN has never been provided, usually the chronologic coherence of MN and malignancy is accepted as a surrogate indicator of the associative link between the two diseases.

MN is histologically characterized by immune deposits on the subepithelial side of the glomerular basement membrane, containing IgG, antigen, and complement molecules. A number of immunohistological studies have shown that IgG4 is the predominant IgG subclass in the glomerular immune deposits in primary MN, leading to the assumption that IgG4 antibodies may induce disease (6, 7). In contrast, the absence of IgG4 and the presence of IgG1 and IgG2 in renal biopsies are considered to be characteristic for secondary, malignancy-associated MN (8–10). Positivity for IgG4 in renal biopsies is common for patients with PLA_2_R1-associated MN and THSD7A-associated MN (11–15), however, in a recent study we did not find any difference in the glomerular IgG1 or IgG4 deposition between patients with THSD7A-associated MN who had, or did not have a malignancy (12). Moreover, findings on glomerular IgG subclass deposition in patients with primary or tumor-associated MN are inconsistent and not confirmed throughout the literature (16). Why these inconsistencies exist is unclear but glomerular stage of disease or use of different staining protocols may play a role (14, 17). Recently we presented data from two patients with THSD7A-expressing tumors and THSD7A-associated MN indicating a possible molecular link between malignancy and autoantibody formation, a mechanism congruent with an autoimmune pathogenesis of malignancy-associated MN (12, 18).

The aim of this study was to investigate potential differences in circulating antigen-specific IgG subclasses between patients with primary and malignancy-associated MN. A systematic approach using Western blot was chosen in order to avoid eventual inconsistencies in renal biopsy findings, allow follow-up measurements of IgG subclass distribution and eventually lead to a better understanding of the pathogenesis of disease. Since IgG4 autoantibodies are considered pathogenetically relevant, we also measured antigen-specific pan-IgG and IgG4-antibody levels in our patient cohort. Moreover, we investigated the binding capacities of the different antigen-specific IgG subclasses in different organs applying human glomerular and lung tissue extracts.

## Materials and Methods

### Patient Cohort and Study Design

In this study 41 consecutive patients with biopsy-proven THSD7A-associated MN were included. The PLA_2_R1-associated MN cohort included 76 patients out of a 302-patients prospective cohort, which we have reported before (12). Since the major objective of the present study was to investigate patients with malignancy-associated MN, we included in this study all 16 (5.3%) out of the 302 PLA_2_R1 antibody (PLA_2_R1-ab) positive patients, who were diagnosed with a malignancy within 24 months of the MN diagnosis. In addition, we included 60 PLA_2_R1-ab positive patients based on the following criteria: (a) 20 patients with persistent PLA_2_R1-ab during follow-up, (b) 20 patients who became negative for PLA_2_R1-ab during follow-up and had no relapse, and (c) 20 patients who became PLA_2_R1-ab negative, but developed a relapse of PLA_2_R1-ab during follow-up.

The study was approved by the local ethics committee of the chamber of physicians in Hamburg and was conducted in accordance with the ethical principles stated by the Declaration of Helsinki. An informed consent was obtained from all participating patients.

### Quantification of IgG Subclass Levels and Antigen Specific IgG4 and Pan IgG

IgG subclass levels (IgG1-4) were measured using the SPAplus Assay from Bindingsite (Product Codes: IgG1 NK006.S, IgG2 NK007.S, IgG3 LK008.S, and IgG4 LK009.S) according to the manufacturer's instructions. Antigen specific pan-IgG and IgG4 were quantified by immunofluorescence test (IFT) as decribed before (12). A monoclonal anti-human IgG4–FITC antibody produced in mouse was used as secondary antibody for measurement of antigen-specific IgG4 (Sigma-Aldrich, F9890). In all patients PLA_2_R1-ab were also measured by ELISA as described before (12).

### Lung and Kidney Tissue Samples

Healthy parts of kidneys from patients who underwent nephrectomy because of a renal tumor were used for the preparation of the human glomerular extracts (HGE) as described before (2). Preparation of lung tissue was performed according to a similar procedure. Healthy parts of lung tissue, which had to be removed during tumor surgery, were used for the preparation of lung tissue extract (LTE). LTE was gathered after sieving of lung tissue using a 150 μm mesh. The tissue fraction >150 μm was homogenized using a Mixer Mill MM400 (Retsch, Haan, Germany) and used for further analyses, while the flow-through was collected to confirm the absence of the antigens. Membrane fractions of both glomeruli and lung tissue were solubilized in a RIPA buffer and contaminating immunoglobulin G was removed using Protein G resin, according to the manufacturer's protocol (GenScript, Piscataway, NJ, USA). Samples for recombinant protein, LTE or HGE were each gathered from one pool of protein/tissue for all performed experiments in the study.

### Measurement of PLA_2_R1-ab and THSD7A-ab and Their Subclasses

Western blot analyses were largely performed according to a protocol previously described (12), representative blots are shown in Supplemental Figure 1. In detail, for detection of PLA_2_R1- and THSD7A-ab by Western Blot, we used the respective recombinant proteins, HGE and LTE. Recombinant PLA_2_R1 and THSD7A were produced in HEK293 cells upon transfection with the respective cDNA as described before (2). Western blot lanes were loaded with the following protein samples: 2 μg of HEK293 cell lysate containing recombinant PLA_2_R1, 0.5 μg HEK293 cell lysate containing recombinant THSD7A, 1 μg of HGE and 6 μg of LTE per Western blot lane. Proteins were separated by electrophoresis in SDS-gels containing 5% acrylamide under non-reducing conditions, unless otherwise specified. Proteins were then transferred to methanol-activated PVDF membranes under semi-dry conditions using Transblot Turbo (Bio-Rad) at 25 V constant voltage for 25 min. Membranes were blocked in 5% dry milk with PBS-Tween (PBS-T, 0.1%) for 1 h at RT and then incubated in primary antibodies at 4°C overnight. Unless otherwise specified, all sera were diluted 1:100 in 0.5% dry milk in PBS-T. For specific detection of the PLA_2_R1 and THSD7A antigens in the positive control of Western blot analyses the following commercially available antibodies were used: rabbit polyclonal antibody at a 1:1000 dilution in 0.5% dry milk in PBS-T (Atlas Antibodies) for PLA_2_R1; rabbit polyclonal antibody at a 1:1,000 dilution in 0.5% dry milk in PBS-T (Atlas Antibodies) for THSD7A. A horseradish peroxidase–conjugated goat anti-rabbit IgG (Sigma-Aldrich, #A9169) was used as secondary antibody at a 1:20,000 dilution. For detection of subclass-specific IgG in Western blot analyses, horseradish peroxidase-conjugated monoclonal anti-human IgG1 (9054-05), IgG2 (9060-05), IgG3 (9210-05), IgG4 (9200-05), and IgG (9040-05) antibodies from mouse were used (IgG1, IgG2, and IgG3 at a 1:5,000 dilution; IgG4 at a 1:10,000 dilution; IgG at a 1:20,000 dilution, all SouthernBiotech, Birmingham, USA), diluted in 5% dry milk in PBS-T.

### Deglycosylation of PLA_2_R1 and THSD7A

HGE, LTE and the respective recombinant proteins were deglycosylated using neuraminidase, *N*-glycopeptidase F and *O*-glycopeptidase (Roche Diagnostics, Mannheim, Germany). One μL of the enzyme solution was added to approximately 20 μl of tissue extract or recombinant protein and samples were incubated for 3 h at 37°C and subsequently analyzed by Western blot.

### Immunofluorescence for PLA_2_R1, THSD7A, and IgG

For immunofluorescence staining of PLA_2_R1 and THSD7A 1 μm paraffin sections of kidney or lung tissue were deparaffinized and rehydrated with water. Antigen retrieval was obtained by boiling in citrate buffer pH 6.1 (both 15 min at constant 120°C). Unspecific binding was blocked with 5% horse serum (Vector) in phosphate-buffered saline (PBS) for 30 min at RT prior to incubation at 4°C o/n with primary antibodies (PLA_2_R1: Atlas, rabbit primary antibody, 1:100; THSD7A: Atlas, mouse primary antibody, 1:100) in blocking buffer. Staining was visualized with fluorochrome-conjugated secondary antibodies (all affinity-purified from Jackson Immunoresearch Laboratories, 1:200) for 10 min RT in 2.5% horse serum in PBS. Nuclei were counterstained with DRAQ5 (Molecular Probes, 1:4,000). All negative controls were performed by omitting primary antibodies. Staining was evaluated with a confocal LSM 800 meta microscope using the LSM software (all Zeiss).

### Statistical Analysis

Remission of proteinuria was defined as proteinuria of <3.5 g/24 h and at least 50% reduction from the time of inclusion. Complete remission of proteinuria was defined as proteinuria <0.5 g/24 h. Continuous data are given as median and 1st−3rd quartile (Q1–Q3), categorical data as counts and percentages (%). Mann-Whitney *U* tests were performed to assess group differences for continuous and ordinal variables. For analyses of dichotomous data, Fisher's exact tests were performed. Statistical significance was defined as *p* < 0.05, two-tailed. Glomerular lesions were assessed by electron microscopy according to the classification of Ehrenreich and Churg (17).

## Results

### Patient Baseline Characteristics

We included 41 consecutive patients with THSD7A-associated MN and 76 patients with PLA_2_R1-associated MN in the study (Table 1 and Supplemental Table 1). Data from some of those patients have been published in an earlier manuscript (12). Patients with THSD7A-associated MN were older than patients with PLA_2_R1-associated MN. Advanced stages of glomerular lesions were more common in patients with PLA_2_R1-associated MN. Differences in other clinical characteristics between the two patient groups were not statistically significant.

**Table 1:** Clinical baseline characteristics of patients included in the study.

		**Complete cohort**	**PLA_**2**_R1-associated MN**	**THSD7A-associated MN**	***P*-value**
Number of patients		117	76	41	
Age–years (median, Q1–Q3)		61, 49–69	59, 45–69	65, 55–75	0.03
Glomerular lesions in renal biopsies (EM) (*n* = 104)	Stage I (%)	17 (16%)	5 (7%)	12 (38%)	<0.01
	Stage II (%)	59 (57%)	43 (60%)	16 (50%)	
	Stage III (%)	15 (14%)	11 (15%)	4 (13%)	
	Stage IV (%)	13 (13%)	13 (18%)	0 (0%)	
PLA_2_R1-ab level, Total-IgG ELISA U/ml (median, Q1–Q3)		80, 2–170	145, 88–313	1, 1–2	<0.01
PLA_2_R1-ab titer, Total-IgG IFT (median, Q1–Q3)		320, 0–3200	1000, 320–10000	0, 0–0	<0.01
PLA_2_R1-ab titer, IgG4-specific IFT (median, Q1–Q3)		10, 0–100	100, 10–100	0, 0–0	<0.01
THSD7A-ab titer, Total-IgG IFT (median, Q1–Q3)		0, 0–100	0, 0–0	320, 100–1000	<0.01
THSD7A-ab titer, IgG4-specific IFT (median, Q1–Q3)		0, 0–10	0, 0–0	10, 32–100	<0.01

*Only parameters with statistically significant difference are presented. The complete list of baseline characteristics is shown in Supplemental Table 1. Q1–Q3**–**1st quartile to 3rd quartile, EM–electron microscopy, PLA_2_R1-ab–PLA_2_R1-antibody, and THSD7A-ab–THSD7A-antibody*.

### Patients With THSD7A-Associated MN

In 30 (73%) patients with THSD7A-associated MN study enrolment and first serum collection for THSD7A-antibody (THSD7A-ab) measurement were performed within 6 months of renal biopsy. In 30 (73%) patients no immunosuppressive treatment was started prior to study enrolment. In seven (17%) patients with THSD7A-associated MN a malignant disease was diagnosed within 2 years of diagnosis of MN (Tables 2, 3 and Supplemental Table 2). The total IgG4 levels were higher in patients with malignancy, compared to patients with no malignancy (0.70 vs. 0.25 g/l), however, the difference did not reach statistical significance (*p* = 0.06) and, moreover, the THSD7A-specific IgG4 levels were not different between the two groups (*p* = 0.65). Otherwise there were no statistically significant differences in the clinical or morphological characteristics of patients with THSD7A-associated MN with or without malignancy. For 25 patients follow-up sera for THSD7A-ab analyses were available. THSD7A-ab became negative in 10 (40%) patients and persisted in 15 (60%) patients (Supplemental Table 3). In patients who became negative for THSD7A-ab immunosuppression was more often used and remission of proteinuria was more common, but the difference was not statistically significant (*p* = 0.09). Doubling of serum creatinine levels occurred at the same rate in both groups, although patients with persistent THSD7A-ab had a better preserved renal function at baseline (not statistically significant, *p* = 0.09). Levels of all IgG subclasses were lower in patients with persistent antibodies, however, none of the differences was statistically significant.

**Table 2:** Clinical baseline characteristics of patients with and those without malignancy.

		**THSD7A-associated MN**		***P*-value**	**PLA**_****2****_**R1-associated MN**		***P*-value**
		**No malignancy**	**Malignancy**		**No malignancy**	**Malignancy**	
Number of patients		34	7		60	16	
Age–years (median, Q1–Q3)		66, 56–75	65, 48–71	0.51	55, 45–65	67, 62–70	0.01
Serum creatinine–mg/dl (median, Q1–Q3)		1.4, 0.9–1.7	1.0, 0.8–1.6	0.42	1.0, 0.8–1.3	1.3, 1.0–2.1	0.04
eGFR–CKD-EPI–mL/min/1.73 m^2^ (median, Q1–Q3)		47, 37–87	75, 50–93	0.35	78, 56–98	53, 35–74	0.01
Tubulointerstitial fibrosis in renal biopsies (*n* = 112)	Minor (< 15%) (%)	20 (69%)	6 (86%)	0.37	41 (68%)	6 (38%)	0.02
	Moderate (16–49%) (%)	8 (28%)	1 (14%)		16 (27%)	8 (50%)	
	Extended (>50%) (%)	1 (3%)	0 (0%)		3 (5%)	2 (13%)	

*Only parameters with statistically significant difference are presented. The complete list of baseline characteristics is shown in Supplemental Table 2. Q1–Q3–1st quartile to 3rd quartile, EM–electron microscopy, PLA_2_R1-ab–PLA_2_R1-antibody, and THSD7A-ab–THSD7A-antibody*.

**Table 3:** Tumor entities diagnosed in patients with malignancy-associated MN.

**Cancer entity**	**Autoantigen**	**Number of patients**	Time between MN diagnosis to tumor diagnosis–months^*^
Hematologic	PLA_2_R1	5	−4, −3, 0, 3, 12
Colon	PLA_2_R1	2	−10, 0
Lung	PLA_2_R1	2	1, 3
Skin, nonmelanoma	PLA_2_R1	2	2, 13
Stomach/esophagus	PLA_2_R1	1	−13
Melanoma	PLA_2_R1	1	−10
Renal cell carcinoma	PLA_2_R1	1	0
Prostate	PLA_2_R1	1	22
Penis	PLA_2_R1	1	23
Colon	THSD7A	2	1, 4
Stomach/esophagus	THSD7A	2	0, 3
Lung	THSD7A	1	0
Prostate	THSD7A	1	19
Uterus	THSD7A	1	0

* *Negative values point out that diagnosis of malignancy was made prior to diagnosis of MN. 17 out of the 23 patients who had a tumor received immunosuppression in addition to tumor treatment*.

### Patients With PLA_2_R1-Associated MN

All 76 patients with PLA_2_R1-associated MN had a newly diagnosed, biopsy-proven MN and were enrolled in a prospective cohort of patients with PLA_2_R1-associated MN. Study enrolment and the first serum collection for PLA_2_R1-antibody (PLA_2_R1-ab) measurement were performed within 6 months of renal biopsy. None of these patients had received an immunosuppressive treatment prior to study enrolment.

The aim of the present study was the identification of subclass differences in malignancy-associated MN compared to primary MN. To this end, all 16 (5.3%) patients, who had a diagnosis of malignancy made within 2 years of MN diagnosis and who participated in the aforementioned prospective cohort of 302 patients with PLA_2_R1-associated MN, were included in this analysis. Patients with PLA_2_R1-associated MN and no malignancy were significantly younger, had better preserved renal function and had less tubulointerstitial fibrosis compared to PLA_2_R1-ab positive patients with malignancy (Table 2 and Supplemental Table 2). Proteinuria was higher in patients with malignancy (not statistically significant, *p* = 0.19), while neither total-IgG PLA_2_R1-ab levels nor IgG4 subclass PLA_2_R1-ab significantly differed between patients with and those without malignancy.

In addition to the 16 PLA_2_R1-ab positive patients with malignancy, we included further 60 patients from our prospective cohort of patients with PLA_2_R1-associated MN in this study. These patients were chosen to reflect a balanced selection of the cohort regarding their development of antibody level during follow-up: (a) 20 patients with persistent PLA_2_R1-ab throughout the study follow-up, (b) 20 patients with stable remission of PLA_2_R1-ab during follow-up (no relapse), and (c) 20 patients with PLA_2_R1-ab relapse.

Patients who became negative for PLA_2_R1-ab during follow-up achieved significantly more often a remission of proteinuria (*p* < 0.01, Supplemental Table 3). They also had 48% lower antibody levels at the beginning of the study, however, this difference was not statistically significant (*p* = 0.12). At baseline we found no statistically significant differences in any of the clinical characteristics between patients, in whom PLA_2_R1-ab relapsed during follow-up, and patients who remained PLA_2_R1-ab negative (Supplemental Table 4). While the rate of initial remission of proteinuria (upon negativity of PLA_2_R1-ab) was not different between the two groups, patients in whom PLA_2_R1-ab relapsed received more often immunosuppressive treatment and had more often a relapse of proteinuria during follow-up (*p* < 0.01 for both).

### Antigen-Specific IgG Subclasses at Baseline Do Not Correlate With the Diagnosis of Malignancy or Clinical Outcome

**Figure 1:**
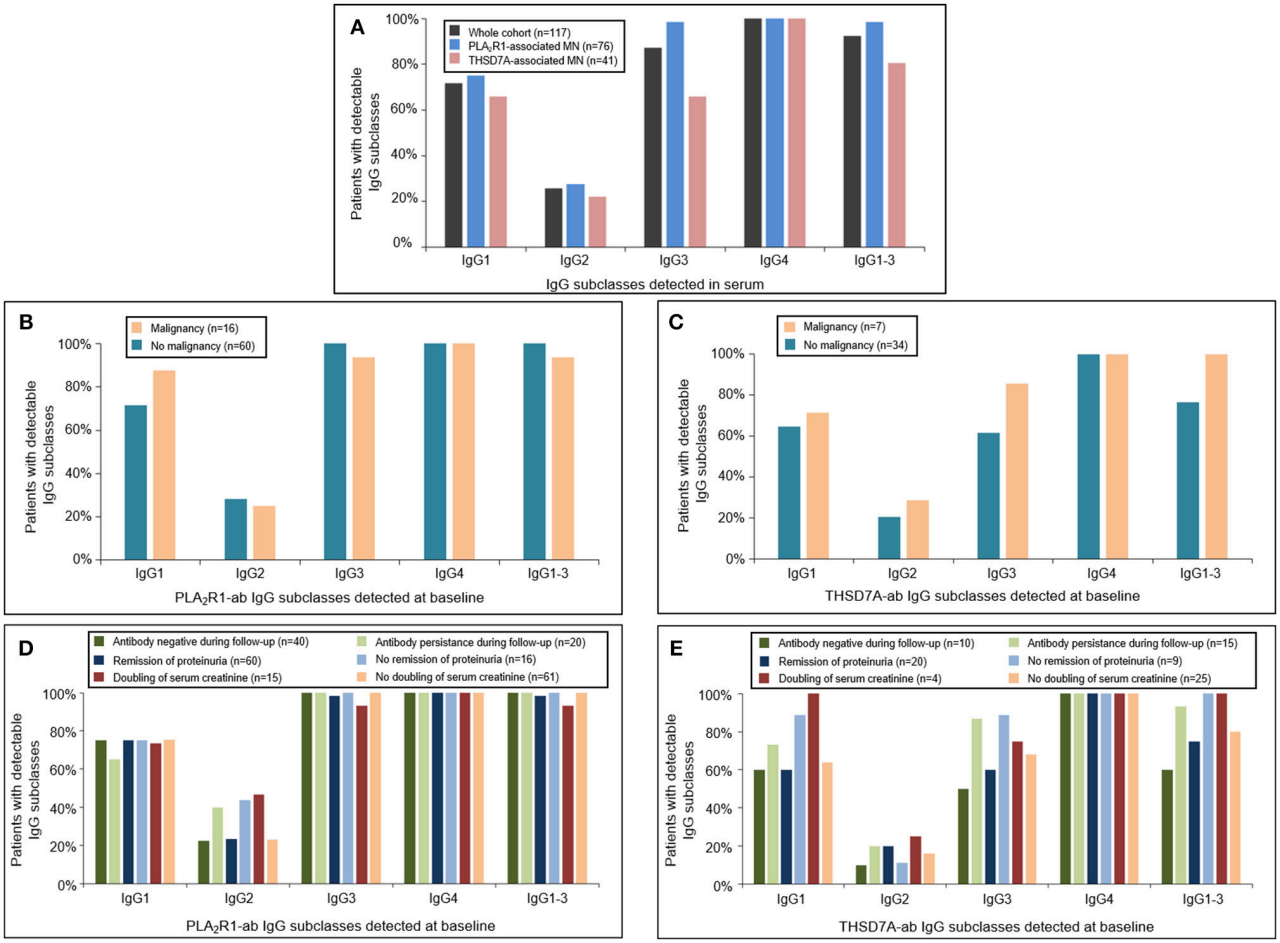
Distribution of PLA_2_R1- and THSD7A-specific IgG subclasses at baseline. **(A)** Detection of antigen-specific IgG subclasses in the complete study cohort by Western Blot using recombinant proteins. All patients were found to be positive for antigen-specific IgG4-antibodies. Antigen-specific IgG3 antibodies were more common than antigen-specific IgG1, while IgG2 was detected less often. The distribution of **(B)** PLA_2_R1- and **(C)** THSD7A-specific IgG subclasses was not different in patients with or without malignancy. Patients with **(D)** PLA_2_R1- or **(E)** THSD7A-associated MN were grouped depending on the clinical outcome: negativity of autoantibodies during follow-up, remission of proteinuria, doubling of serum creatinine. IgG1-3 depicts the percentage of patients in whom at least one antigen-specific IgG subclass (IgG1, IgG2, or IgG3) was detected in addition to IgG4.

At baseline all patients had antigen-specific IgG4 subclass antibodies directed against PLA_2_R1 or THSD7A (Figure 1). Antigen-specific IgG3 subclass was detected in almost all PLA_2_R1-ab positive patients (99%), followed by IgG1 (75%) and IgG2 subclass (28%). In THSD7A-ab positive patients antigen-specific IgG3 and IgG1 subclass were present in the same number of patients (66%), while IgG2 was less common and found in 22% of the THSD7A-associated cases. IgG subclass patterns did not differ significantly between THSD7A-ab positive patients with or without immunosuppressive treatment prior to study inclusion.

**Figure 2:**
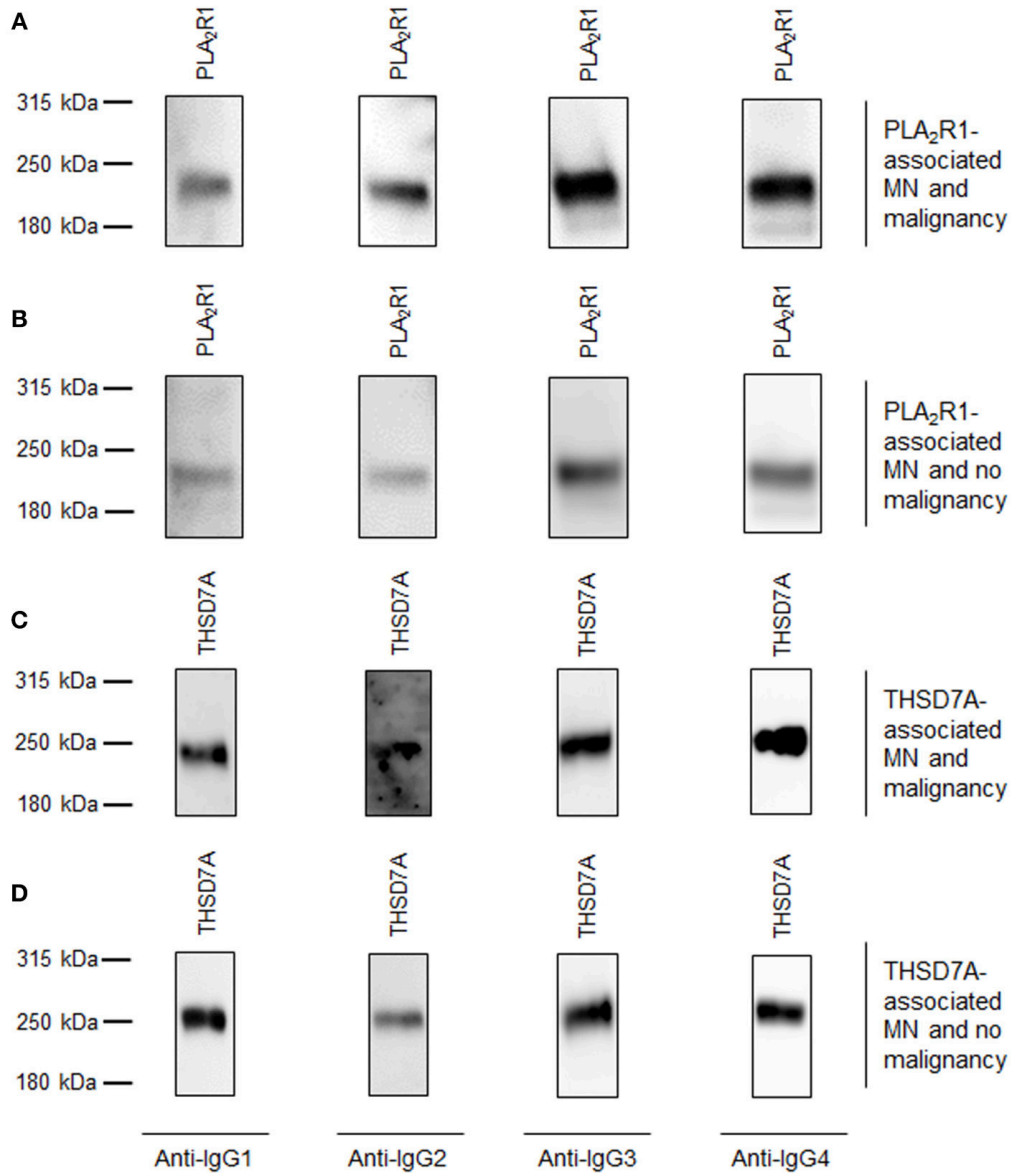
Representative Western blots of patients with primary MN and malignancy-associated MN. Representative Western blot analyses of patients with all 4 subclasses of antigen-specific IgG binding. **(A)** Patient 1 with PLA_2_R1-associated MN and malignancy. **(B)** Patient 2 with PLA_2_R1-associated MN and no malignancy. **(C)** Patient 3 with THSD7A-associated MN and malignancy. **(D)** Patient 4 with THSD7A-associated MN and no malignancy.

We did not detect any statistically significant differences in antigen-specific IgG subclasses between primary and malignancy-associated MN (Table 2, Supplemental Table 2, Figures 1B,C, 2). Antigen-specific antibodies of IgG4 subclass were found in all patients in both groups, irrespective of the antigen. In PLA_2_R1-ab positive patients, antigen-specific IgG1, IgG2, and IgG3 antibodies were positive in 72 vs. 88%, 28 vs. 25%, and 100 vs. 94% of patients with primary vs. malignancy-associated MN, respectively (Figure 1B). In THSD7A-ab positive patients antigen-specific IgG1, IgG2, and IgG3 were found in 65, 21, and 62% of cases with primary MN vs. 71, 29, and 86% of cases with malignancy-associated MN, respectively (Figure 1C). Seventeen out of the 23 patients with MN and malignancy received an immunosuppressive treatment in addition to the treatment of malignancy. Ten of these patients had a remission of proteinuria (five complete remission and five partial remission), five patients had no remission of proteinuria and two patients died during follow-up. Therefore, conclusive evidence on the effect of tumor treatment on disease development is not possible. Three out of six patients who received no immunosuppressive treatment had a remission of proteinuria (two complete remission, one partial remission), one patient had no remission while no follow-up data were available for the remaining two patients.

We next grouped patients with PLA_2_R1- or THSD7A-associated MN depending on the clinical outcome (antibody negative during follow-up, remission of proteinuria, doubling of serum creatinine) and analyzed the respective IgG subclass patterns at baseline. There were no statistically significant differences in the IgG subclass patterns for PLA_2_R1-ab (Figure 1D) or THSD7A-ab (Figure 1E) at baseline, which might discriminate patients with a favorable from those with an unfavorable outcome.

### Quantification of Antigen-Specific IgG4

Since IgG4 is assumed to play a central pathogenic role in primary MN and to differentiate primary from malignancy-associated MN, we deeper analyzed the IgG4-specific findings in our cohort. 100% of the patients with PLA_2_R1- or THSD7A-associated MN showed presence of antigen-specific IgG4 antibodies, irrespective of the presumed pathogenesis. We next measured the antigen-specific IgG4 levels in all patients and found no statistically significant difference between the antigen-specific pan-IgG, or IgG4 levels in patients with malignancy and those with no malignancy (Tables 1, 2). Furthermore, serum levels of all IgG subclasses were not statistically different between these patient groups.

### Antigen-Specific IgG Subclasses During Follow-Up

**Figure 3:**
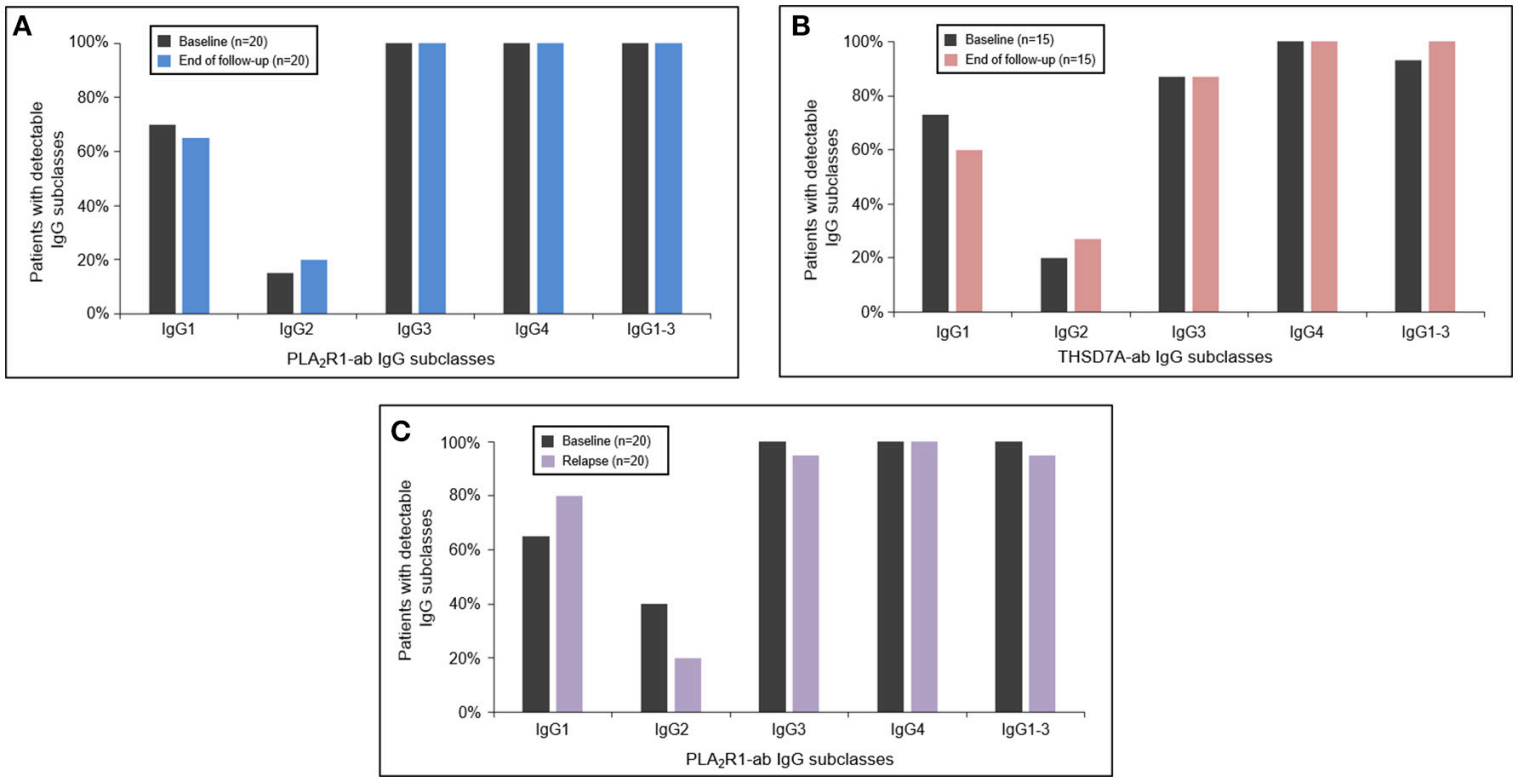
Antigen-specific IgG subclasses during follow-up and relapse. The PLA_2_R1- **(A)** and THSD7A-specific **(B)** IgG subclasses did not significantly change during the follow-up period. At the time of relapse of PLA_2_R1-ab, antigen-specific IgG subclasses were similar to the distribution observed at baseline **(C)**.

In 20 patients with PLA_2_R1-associated MN and 15 patients with THSD7A-associated MN autoantibodies persisted (antibodies were never negative in IFT or ELISA analysis) throughout the median study follow-up period of 15 months (Q1–Q3: 12–22 months) and 15 months (Q1–Q3: 12–27 months), respectively (Supplemental Table 3). In these patients there were no significant changes in any of the antigen-specific IgG subclasses between baseline and the end of the study follow-up (Figures 3A,B). In 20 PLA_2_R1-ab positive patients autoantibodies relapsed after a median follow-up time of 21 months (Q1–Q3: 14–40 months) (Supplemental Table 4). At the time of relapse, PLA_2_R1-specific IgG subclasses were not significantly different from the findings at baseline (Figure 3C).

### PLA_2_R1 and THSD7A Expression in Human Tissues

**Figure 4:**
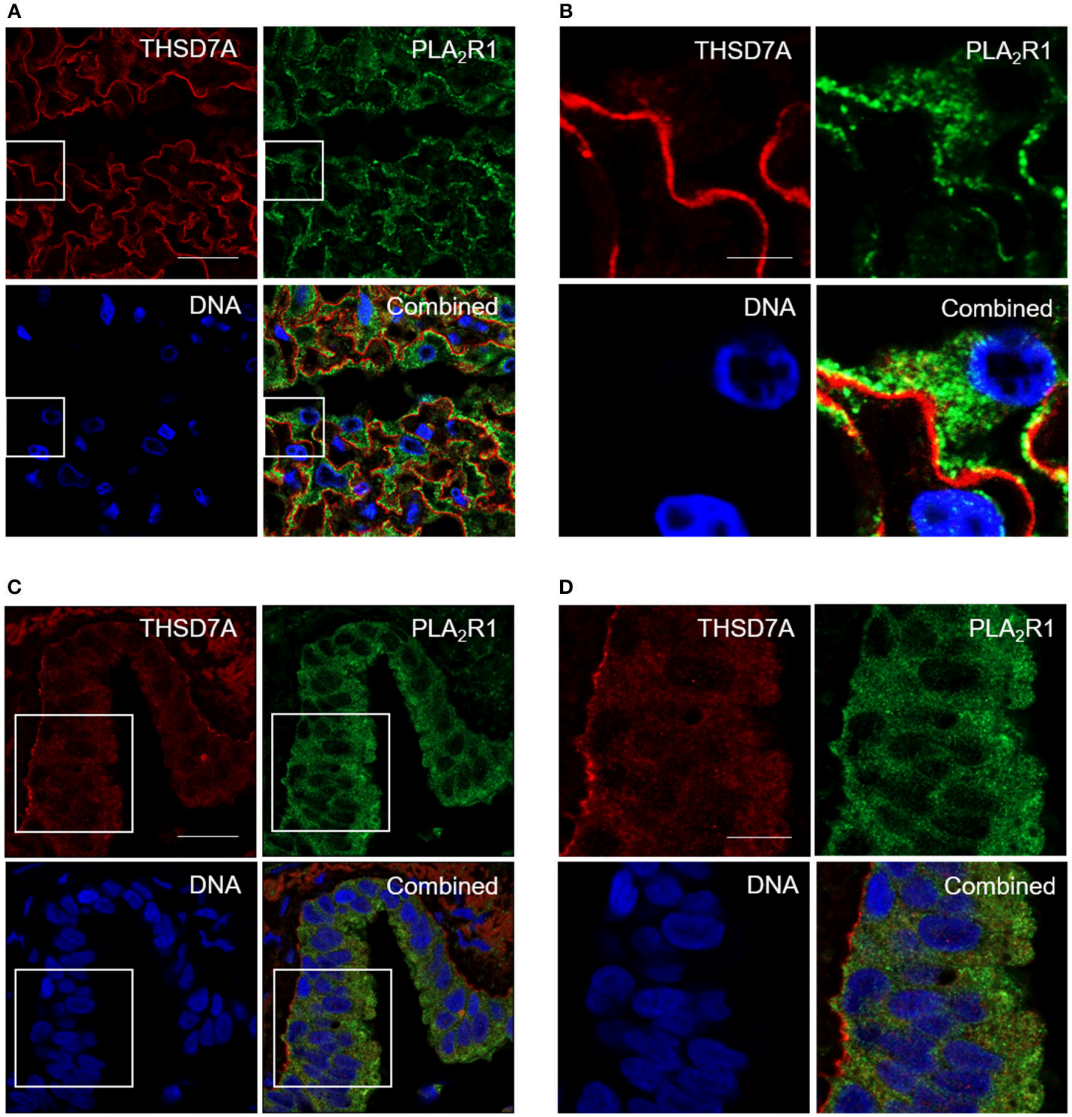
Immunofluorescence staining of human kidney and lung tissue for PLA_2_R1 and THSD7A. Immunofluorescence staining of kidney **(A,B)** and lung **(C,D)** tissue showed positivity for human PLA_2_R1 (red) and THSD7A (green). PLA_2_R1 and THSD7A showed only discreet overlap in the glomerulus and no overlap in the lung. Nuclei were counterstained using DRAQ5 (DNA: blue). Scale bars: 20 μm **(A,C)**, 5 μm **(B)**, and 10 μm **(D)**.

In order to confirm whether these IgG subclass binding patterns are specific for the kidney, or may also apply to other organs, we searched for another human tissue where both antigens are stably expressed. Using immunofluorescence staining we, in fact, found that PLA_2_R1 as well as THSD7A were expressed not only in podocytes, but also lung bronchioles (Figure 4). In human glomeruli PLA_2_R1 was detected in the intracellular compartment and on the surface of podocytes, whereas THSD7A is expressed in an almost linear pattern at the basolateral side of the podocyte. In lung bronchioles PLA_2_R1 is located within the epithelial cells, whereas THSD7A was also found at the basolateral side of the cells. When analyzed by confocal microscopy, PLA_2_R1 and THSD7A showed only discreet overlap in the glomerulus, while no overlap was detected in the bronchioles (Figure 4, Supplemental Figure 2).

**Figure 5:**
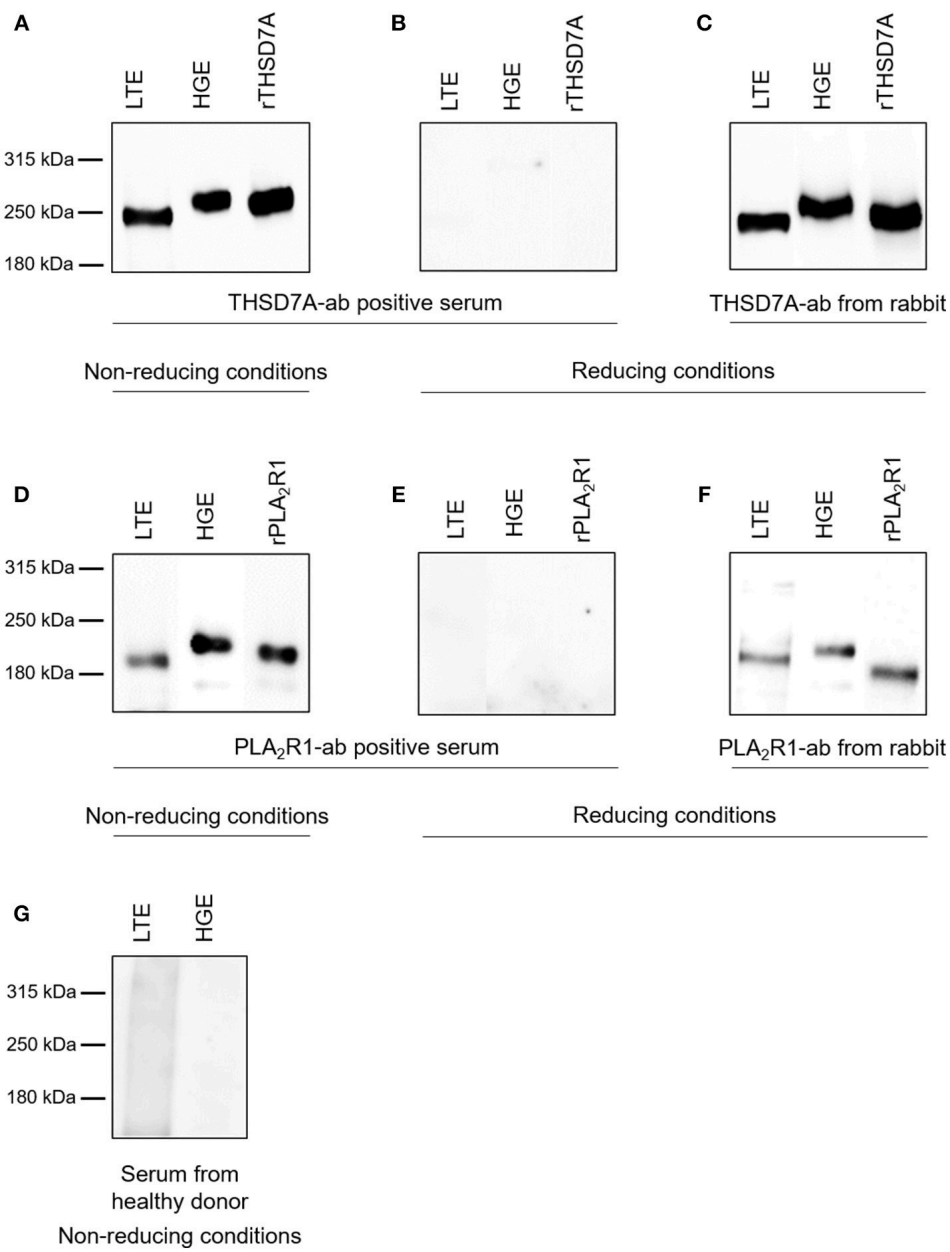
Detection of PLA_2_R1 and THSD7A in human glomerular and lung tissue. THSD7A was detectable in both lung tissue extracts (LTE) and human glomerular extracts (HGE) when analyzed with serum from a patient with THSD7A-ab positive MN under non-reducing conditions **(A)**. The positivity for THSD7A was completely abolished when Western blot analyses were performed under reducing conditions **(B)**. THSD7A detection in both tissues was confirmed using a commercially available THSD7A-ab from rabbit **(C)**. The same analyses were performed for detection of PLA_2_R1 in LTE and HGE using serum from a patient with PLA_2_R1-ab positive MN **(D,E)** or a commercially available PLA_2_R1-ab from rabbit **(F)**. Serum from a healthy donor failed to detect either of the antibodies in both tissues **(G)**.

**Figure 6:**
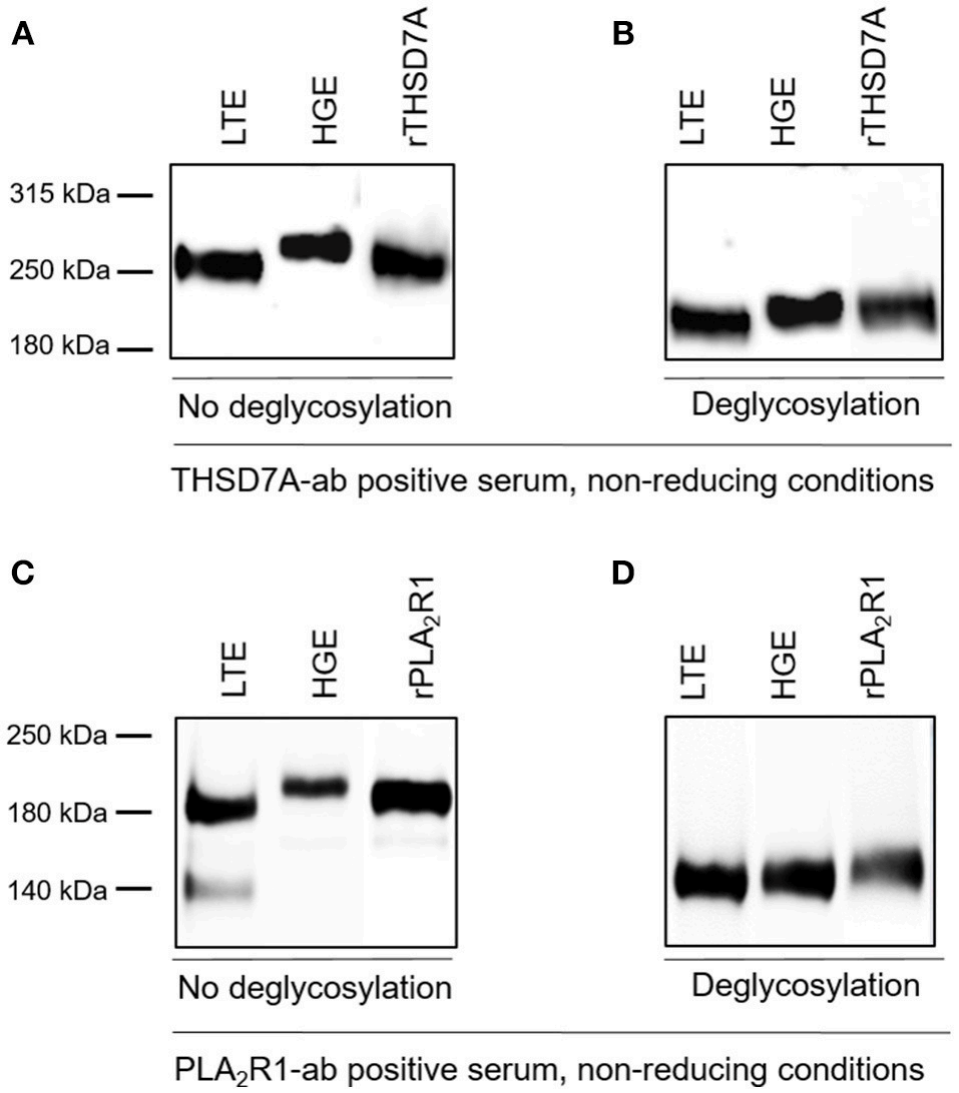
THSD7A and PLA_2_R1 are differentially glycosylated in human lung and kidney. **(A)** The THSD7A-ab positive serum shows positive binding to the antigen of human lung tissue (LTE) and glomerular extract (HGE), however, the antigen migrates differentially in both tissues. The renal antigen showed a higher molecular weight compared to the lung antigen. **(B)** Upon addition of deglycosylating enzymes THSD7A shows the same migration pattern in both tissues, suggesting a polypeptide backbone with different glycosylation in the lung and the kidney. **(C,D)** Similar observations were made for PLA_2_R1 in lung and kidney tissue. 140 kDa band in LTE represents residual IgG, which was also detected in negative controls (healthy serum, *n* = 5, data not shown).

The presence of PLA_2_R1 and THSD7A in lung tissue was further confirmed by Western blot analyses (Figure 5). In both cases the antigen-antibody interaction was only detected under non-reducing conditions, suggesting that the antibodies bound to the conformational structures of the antigens in both organs. We observed a different migrating pattern of both antigens derived from the lung and the kidney, which was abolished after deglycosylation, indicating tissue-specific differences in antigen glycosylation for both PLA_2_R1 and THSD7A (Figure 6).

### Binding Characteristics of PLA_2_R1 and THSD7A Antibody Subclasses to Glomerular and Lung Tissue Extracts

**Figure 7:**
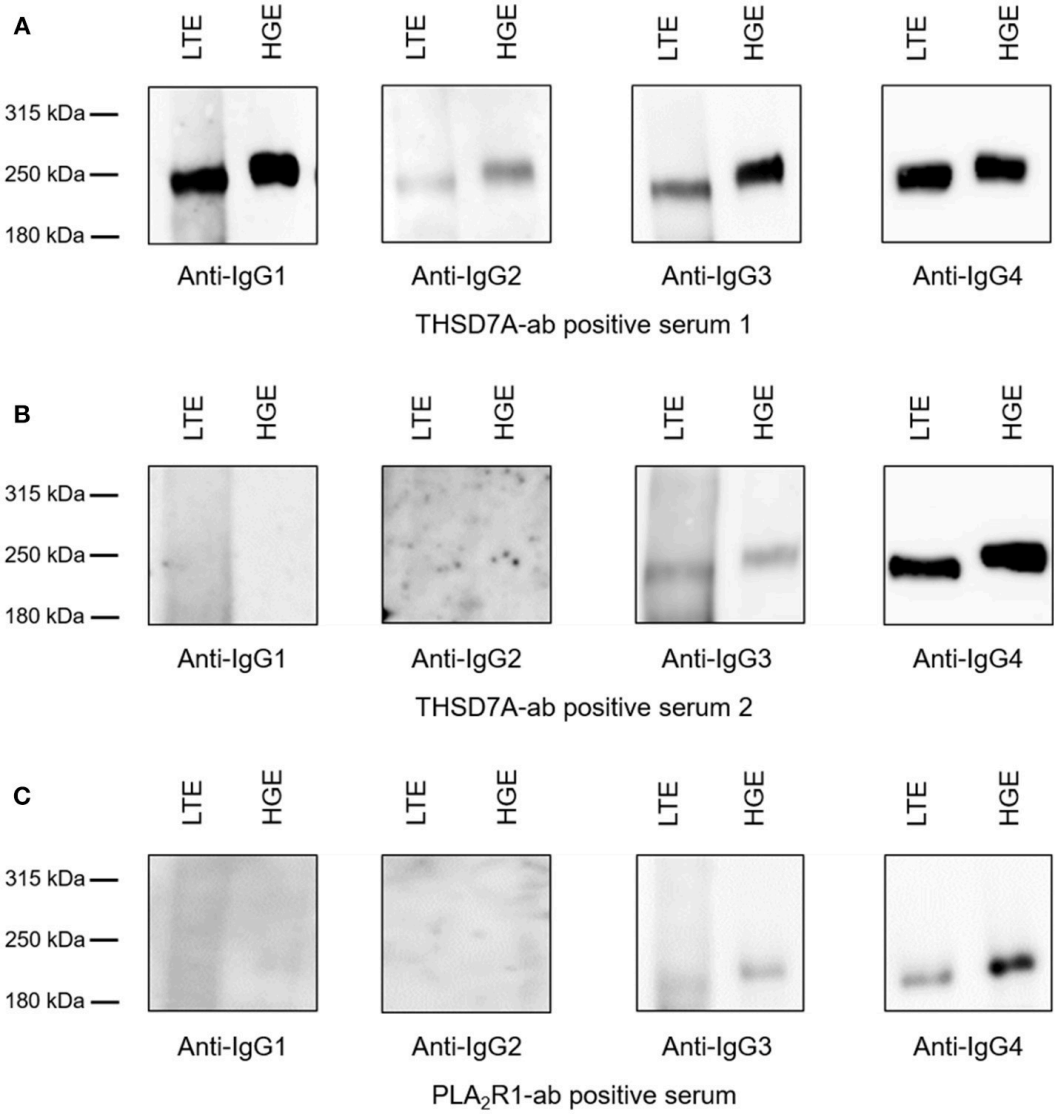
Binding of antigen-specific IgG subclasses to human glomerular and lung tissue. Representative Western blot analyses of the antigen-specific IgG subclasses binding to lung tissue extracts (LTE) and human glomerular extracts (HGE). **(A)** Serum from patient 1 was positive for all THSD7A-specific IgG subclasses. **(B)** Serum from patient 2 showed detectability for THSD7A-specific IgG3 and IgG4. **(C)** Serum from patient 3 was positive for PLA_2_R1-specific IgG3 and IgG4.

Binding of all antigen-specific IgG subclasses to glomerular and lung tissue was investigated for all THSD7A-ab positive patient sera at baseline (*n* = 41) as well as all 15 sera with persistent THSD7A-ab positivity during follow-up. All analyzed sera showed binding to glomerular and lung THSD7A antigen under non-reducing conditions and presented identical IgG subclass patterns in the kidney and the lung (Figure 7). To confirm these findings in patients with PLA_2_R1-associated MN, we performed the analyses in a selection of PLA_2_R1-ab positive sera at baseline: a) three patients, in whom PLA_2_R1-ab had disappeared during follow-up, b) three patients with PLA_2_R1-associated MN and malignancy, and c) four patients with persistence of PLA_2_R1-ab, in whom we compared sera at baseline and during follow-up. Equivalently to the THSD7A-ab positive sera, all 14 PLA_2_R1-ab positive sera showed identical IgG subclass pattern binding to lung and kidney tissue extracts (Figure 7).

## Discussion

The pathogenesis of primary MN is defined by the binding of circulating antibodies to antigens expressed on podocytes (19). In contrast, the pathophysiology of malignancy-associated MN and the causality link between tumor and MN remain unsolved, although they have been extensively debated since the description of a possible relationship between tumors and the diagnosis of MN more than 50 years ago (20). Usually, an association is assumed based on a defined time course of both diseases (8–10, 12). The immunologic processes leading to primary MN are believed to differ from those inducing malignancy-associated MN, since some studies reported significant differences between IgG subclasses in the renal biopsies of patients with primary MN and patients with malignancy-associated MN. However, published data on the association of IgG4 negativity in renal biopsies of patients with malignancy-associated MN are inconsistent (16). Some studies suggested an association of glomerular absence for IgG4 with malignancy (8, 9), while others did not confirm these findings, but showed an association of glomerular IgG1 and IgG2 with malignancy (10).

Binding of circulating antibodies to endogenous podocyte antigens is assumed to initiate primary MN. In contrast, the deposition of tumor antigens in the subepithelial space with consecutive binding of circulating antibodies or the glomerular deposition of circulating immune complexes containing tumor antigens and antibodies have been considered to be the origin of malignancy-associated MN. This concept was recently challenged by findings in two cases of THSD7A-associated MN with concurrent malignancy (12). These patients revealed pathomechanisms of disease equivalent to the presumed pathogenesis in primary MN: they disposed autoantibodies directed against an endogenous podocyte antigen. This observation of THSD7A-ab positive MN in association with cancer and malignancy is highly reminiscent of paraneoplastic disease. Paraneoplastic syndromes have diverse presentations and their pathomechanisms are often not well-understood. In the kidney, they often present as a glomerulonephritis, suggesting an important role of autoimmune pathomechanisms (20–22).

To better understand potential differences in the immune pathomechanisms of primary vs. malignancy-associated MN we systematically analyzed the binding of circulating antigen-specific IgG subclasses from patients with MN to PLA_2_R1 and THSD7A extracted from human glomeruli. We found antigen-specific IgG4 antibodies in all patients, independent of the presumed pathogenesis of disease. Comparison of antigen-specific IgG subclass patterns in PLA_2_R1- and THSD7A-ab positive patients did not show significant differences between primary MN and malignancy-associated MN. The antigen-specific IgG4 levels also did not significantly differ between patients with primary and malignancy-associated MN. Thus, our data support the hypothesis that the evolution of the humoral response is similar for both the PLA_2_R1 and THSD7A antigen, independently of the diagnosis of a malignancy.

Considering that MN is a rare disease, especially the THSD7A-associated disease, our cohort is reasonably large. However, we might have missed small associations, especially concerning IgG1-3, which might have become visible in even larger cohorts. This is most likely not the case for IgG4, since antigen-specific IgG4 was detected in all patients in the study, irrespective of malignancy-association or autoimmune pathogenesis. In our cohort, patients with PLA_2_R1-associated MN with malignancy were significantly older than those patients without malignancy, which might suggest that a number of tumors might be age-related.

In contrast to immunohistology the detection of IgG subclasses in the serum via Western blot is antigen-specific and unbiased of glomerular disease stage. Therefore, such an analysis of circulating antigen-specific antibody subclasses might be more accurate compared to glomerular deposited IgG. However, we cannot exclude the possibility of differences in the sensitivity of the tests (Western blot vs. immunohistochemistry) or that certain IgG subclasses might have a higher tendency to glomerular deposition. In patients with MN it is unclear, whether the *in situ* glomerular binding of circulating autoantibodies in patients with malignancy and those without malignancy depends only on the antigen-antibody binding properties (affinity, specificity, antibody levels etc.), or whether “local” glomerular factors may also play a role *in vivo*. While an *in vitro* experimental approach is not suitable to conclusively answer this question, our data clearly show, that circulating antigen-specific pan-IgG and IgG4 levels are not different between patients with malignancy-associated MN and those with no malignancy. IgG4-deposition in MN is frequently considered proof of a primary disease pathogenesis. However, in our view it is unlikely that circulating antigen-specific IgG4 molecules (which are found in all patients irrespectively of malignancy) bind more often in the glomeruli of patients without malignancy, compared to patients with malignancy if we consider our findings that the specificity, the antigen-specific IgG4-quantity and the antigen-specific pan-IgG-quantity are not different between patients with and those without malignancy.

Analysis of IgG subclasses deposited in the glomeruli shows only a snapshot of IgG subclasses, i.e., at the time of biopsy, while serum measurement allows serial determination of IgG subclasses during follow-up. In our cohort the composition of the antigen-specific IgG subclasses in the serum did not significantly change over time in patients with persistent autoantibodies. Because of the small patient cohort and the fact that different immunosuppressive treatments were used, our data do not allow a conclusion whether a specific immunosuppressive treatment influences the immune response (i.e., IgG subclasses) differently compared to the other treatments. However, in the cohort as a whole, there were no significant changes in any of the antigen-specific IgG subclasses between baseline and the end of the study follow-up. If patients had a relapse, antigen-specific IgG subclasses at relapse were not significantly different from those at baseline, suggesting a stable repertoire of antibody-producing cells in these patients. These findings are relevant, since IgG4 antibodies show a different ability to activate complement compared to other antibody subclasses. Considering the very variable prognosis of MN, it would be conceivable that the presence of strong complement-activating autoantibodies (i.e., IgG3) might be related to a less favorable prognosis. At the same time, patients presenting with only low-complement-activating autoantibodies (i.e., IgG4) at baseline or during follow-up might have a better prognosis and more often a spontaneous remission of disease. Our data do not support such a hypothesis, since the antigen-specific IgG subclasses were not associated with a better outcome defined as autoantibody depletion, remission of proteinuria, or development of renal insufficiency during follow-up.

The antigen-binding characteristics of the autoantibodies were not specific to the glomeruli, but also applied to the lung, where we demonstrated expression of PLA_2_R1 and THSD7A in close proximity in the bronchioles. All antigen-specific IgG subclasses bound to both antigens in the lung and the kidney only when Western blot was performed under non-reducing conditions, indicating conformational epitopes in both organs. Since SDS-gels were used for the Western Blot analyses and tissue extracts were solubilized in RIPA buffer, one can expect that the antigens have been denatured. However, the experimental conditions were chosen as such, that disulfide bonds would be intact. The detection of the antigens under these conditions suggests that disulfide bonds are required and sufficient for conservation of the conformational epitopes in both antigens. Both proteins showed organ-specific modifications as they were both differentially glycosylated in the glomerulus. Although both antigens are expressed and presented in the lung, MN does usually not clinically affect the lung. One could speculate that anatomic differences of the distinct epithelia could have an impact on antigen accessibility. On the other hand, immune complex clearing could be hampered in the subepithelial space of the glomeruli, whereas bronchial epithelium, being in direct contact to the environment, has a high cell turnover and possibly a facilitated clearing of immune deposits via the sputum.

We used Western blot technique for IgG subclass detection, which has been shown to be the most sensitive method for antibody detection (12). Still, we might have missed detection of certain subclasses in some patients, if autoantibody levels were extremely low, falling below the detectability threshold of Western blot. This could be ruled out in the case of IgG4 (100% positivity in all patients), but might have played a role in the case of IgG2. Our data show no significant differences in the antigen-specific IgG subclass distribution in primary vs. malignancy-associated MN and thus support the view that the pathogenesis of primary and malignancy-associated MN might follow similar immunological principles. Primary antigen recognition may occur in human organs other than the kidney, in malignant tumors (18), or benign tumors (23). By mechanisms which we do not yet understand, tolerance is broken and high affinity antibodies of potentially all subclasses are formed and bind to PLA_2_R1 or THSD7A expressed on podocytes, leading ultimately to MN. Further research including genetic associations and pathogenic epitope profiles in patients with malignancy-associated and primary MN could help elucidate pathomechanisms leading to MN. A number of studies have shown a clear association of specific HLA haplotypes with the incidence of MN (24, 25). These haplotypes differ between different ethnicities and might also differ between PLA_2_R1- and THSD7A-associated MN. Whether different haplotypes may also be associated with different disease pathomechanisms, i.e., malignancy-associated MN vs. primary MN, remains to be elucidated. Since the production of class-switched specific IgG1-4 antibodies (and potential HLA associations) indicate a T cell driven process, a more detailed investigation of the role of T cells in MN development might lead to important findings on the underlying pathomechanisms of disease development. Whether differences in the antigen-specific IgG subclass quantity, especially for autoantibodies directed against different potential pathogenic epitopes play a clinical role, remains an open question. In THSD7A-associated MN the epitope profiles of patients with or without malignancy were not significantly different and did not predict disease outcome (26).

Since we analyzed antigen-specific immunoglobulins, our results are limited to cases with PLA_2_R1- and THSD7A-associated cases. Whether our findings may or may not be transferred to patients with PLA_2_R1-ab and THSD7A-ab negative MN, especially in those patients with malignancies, who show a negative IgG4 staining in the biopsy remains an open question. The identification of new target antigens in MN will eventually help to better understand the pathomechanisms in these cases.

In conclusion, we show that the PLA_2_R1 and THSD7A antibody subclass distribution in the serum is not different in patients with primary MN and those with malignancy-associated disease. These data support the idea that the pathogenesis of primary and malignancy-associated MN might follow a similar pathogenetic principle.

## Author Contributions

EH and RS were responsible for the conception and design of the study. Acquisition of data was performed by FvH and EH. MR, AS, and FvH processed the kidney tissue. HP performed the statistical analyzes. FvH, LR, EH, and RS analyzed and interpreted the data. Drafting and revising of the article was performed by FvH, LR, EH, and RAKS. All authors have approved the final version of the manuscript and agree to be accountable for all aspects of the work.

### Conflict of Interest Statement

The authors declare that the research was conducted in the absence of any commercial or financial relationships that could be construed as a potential conflict of interest.
