# Supplementary material for: Antigen-Specific IgG Subclasses in Primary and Malignancy-Associated Membranous Nephropathy
Source: Front Immunol. 2018 Dec 20;9:3035. doi: 10.3389/fimmu.2018.03035 (PMC6306563; doi:10.3389/fimmu.2018.03035)

**Supplemental Table 1: Clinical baseline characteristics of patients included in the study.**

Q1 – Q3 – 1^st^ quartile to 3^rd^ quartile; * – for 99 patients sufficient amount of serum was available to perform measurement of all IgG-subclass-levels (32 patients with THSD7A-associated MN and 67 patients with PLA_2_R1-associated MN); EM – electron microscopy; PLA_2_R1-ab – PLA_2_R1-antibody; THSD7A-ab – THSD7A-antibody

|  | | **Complete cohort** | **PLA_2_R1-associated MN** | **THSD7A-associated MN** | **P-value** |
| --- | --- | --- | --- | --- | --- |
| **Number of patients** | | 117 | 76 | 41 |  |
| **Age - years (median, Q1 – Q3)** | | 61, 49-69 | 59, 45-69 | 65, 55-75 | 0.03 |
| **Male gender (%)** | | 74 (63%) | 50 (66%) | 24 (59%) | 0.55 |
| **Proteinuria - g/24h (median, Q1 – Q3)** | | 6.9, 4.6-9.7 | 6.9, 5.0-9.8 | 6.6, 4.0-9.6 | 0.24 |
| **Serum creatinine - mg/dl (median, Q1 – Q3)** | | 1.1, 0.9-1.6 | 1.0, 0.9-1.4 | 1.3, 0.9-1.7 | 0.12 |
| **eGFR – CKD-EPI - mL/min/1.73 m^2^ (median, Q1 – Q3)** | | 69, 42-92 | 73, 49-94 | 53, 36-89 | 0.08 |
| **Tubulointerstitial fibrosis in renal biopsies (n=112)** | **Minor (≤15%) (%)** | 73 (65%) | 47 (62%) | 26 (72%) | 0.26 |
|  | **Moderate (16-49%) (%)** | 33 (29%) | 24 (32%) | 9 (25%) |  |
|  | **Extended (≥50%) (%)** | 6 (5%) | 5 (7%) | 1 (3%) |  |
| **Glomerular lesions in renal biopsies (EM) (n=104)** | **Stage I (%)** | 17 (16%) | 5 (7%) | 12 (38%) | <0.01 |
|  | **Stage II (%)** | 59 (57%) | 43 (60%) | 16 (50%) |  |
|  | **Stage III (%)** | 15 (14%) | 11 (15%) | 4 (13%) |  |
|  | **Stage IV (%)** | 13 (13%) | 13 (18%) | 0 (0%) |  |
| **Time from renal biopsy to study inclusion - months (median, Q1 – Q3)** | | 0.8, 0.0-2.0 | 0.8 0.0-1.6 | 0.8 0.3-3.0 | 0.59 |
| **IgG1 level (g/L) (median, Q1 – Q3) (n=99) *** | | 2.37, 1.51-3.26 | 2.29, 1.25-3.14 | 2.83, 1.86-3.71 | 0.14 |
| **IgG2 level (g/L) (median, Q1 – Q3) (n=99) *** | | 1.37, 0.92-2.12 | 1.39, 0.91-2.08 | 1.21, 0.97-2.25 | 0.87 |
| **IgG3 level (g/L) (median, Q1 – Q3) (n=99) *** | | 0.41, 0.27-0.58 | 0.36, 0.27-0.50 | 0.49, 0.27-0.65 | 0.20 |
| **IgG4 level (g/L) (median, Q1 – Q3) (n=99) *** | | 0.25, 0.16-0.46 | 0.24, 0.14-0.41 | 0.29, 0.19-0.69 | 0.15 |
| **PLA_2_R1-ab level, Total-IgG ELISA U/ml (median, Q1 – Q3)** | | 80, 2-170 | 145, 88-313 | 1, 1-2 | <0.01 |
| **PLA_2_R1-ab titer, Total-IgG IFT (median, Q1 – Q3)** | | 320, 0-3200 | 1000, 320-10000 | 0, 0-0 | <0.01 |
| **PLA_2_R1-ab titer, IgG4-specific IFT (median, Q1 – Q3)** | | 10, 0-100 | 100, 10-100 | 0, 0-0 | <0.01 |
| **THSD7A-ab titer, Total-IgG IFT (median, Q1 – Q3)** | | 0, 0-100 | 0, 0-0 | 320, 100-1000 | <0.01 |
| **THSD7A-ab titer, IgG4-specific IFT (median, Q1 – Q3)** | | 0, 0-10 | 0, 0-0 | 10, 32-100 | <0.01 |

**Supplemental Table 2: Clinical baseline characteristics of patients with and those without malignancy.**

Q1 – Q3 – 1^st^ quartile to 3^rd^ quartile; * – for 99 patients sufficient amount of serum was available to perform measurement of all IgG-subclass-levels: 25 patients with THSD7A-associated MN and no malignancy and 7 patients with malignancy as well as 53 patients with PLA_2_R1-associated MN with no malignancy and 14 patients with malignancy); EM – electron microscopy; PLA_2_R1-ab – PLA_2_R1-antibody; THSD7A-ab – THSD7A-antibody.

|  | | **THSD7A-associated MN** | | **P-value** | **PLA_2_R1-associated MN** | | **P-value** |
| --- | --- | --- | --- | --- | --- | --- | --- |
|  |  | **No malignancy** | **Malignancy** |  | **No malignancy** | **Malignancy** |  |
| **Number of patients** | | 34 | 7 |  | 60 | 16 |  |
| **Age - years (median, Q1 – Q3)** | | 66, 56-75 | 65, 48-71 | 0.51 | 55, 45-65 | 67, 62-70 | 0.01 |
| **Male gender (%)** | | 19 (56%) | 5 (71%) | 0.68 | 38 (63%) | 12 (75%) | 0.56 |
| **Proteinuria - g/24h (median, Q1 – Q3)** | | 6.3, 3.9-9.2 | 8.2, 5.5-10.3 | 0.48 | 6.5, 4.7-9.5 | 8.5, 6.7-9.8 | 0.19 |
| **Serum creatinine - mg/dl (median, Q1 – Q3)** | | 1.4, 0.9-1.7 | 1.0, 0.8-1.6 | 0.42 | 1.0, 0.8-1.3 | 1.3, 1.0-2.1 | 0.04 |
| **eGFR – CKD-EPI - mL/min/1.73 m^2^ (median, Q1 – Q3)** | | 47, 37-87 | 75, 50-93 | 0.35 | 78, 56-98 | 53, 35-74 | 0.01 |
| **Tubulointerstitial fibrosis in renal biopsies (n=112)** | **Minor (<15%) (%)** | 20 (69%) | 6 (86%) | 0.37 | 41 (68%) | 6 (38%) | 0.02 |
|  | **Moderate (16-49%) (%)** | 8 (28%) | 1 (14%) |  | 16 (27%) | 8 (50%) |  |
|  | **Extended (>50%) (%)** | 1 (3%) | 0 (0%) |  | 3 (5%) | 2 (13%) |  |
| **Glomerular lesions in renal biopsies (EM) (n=104)** | **Stage I (%)** | 10 (40%) | 2 (29%) | 0.31 | 2 (4%) | 3 (19%) | 0.07 |
|  | **Stage II (%)** | 13 (52%) | 3 (43%) |  | 33 (59%) | 10 (63%) |  |
|  | **Stage III (%)** | 2 (8%) | 2 (29%) |  | 10 (18%) | 1 (6%) |  |
|  | **Stage IV (%)** | 0 (0%) | 0 (0%) |  | 11 (20%) | 2 (13%) |  |
| **Time from renal biopsy to study inclusion – months (median, Q1 – Q3)** | | 0.8, 0.1-5.0 | 0.8, 0.3-1.0 | 0.67 | 0.8, 0.0-1.4 | 0.6, 0.2-1.6 | 0.84 |
| **IgG1 level (g/L) (median, Q1 – Q3) (n=99) *** | | 2.79, 1.82-3.57 | 3.07, 2.67-3.61 | 0.54 | 2.29, 1.31-3.15 | 2.46, 1.28-3.04 | 0.99 |
| **IgG2 level (g/L) (median, Q1 – Q3) (n=99) *** | | 1.13, 0.80-2.18 | 1.72, 1.40-2.30 | 0.28 | 1.57, 0.97-2.11 | 1.08, 0.90-1.77 | 0.27 |
| **IgG3 level (g/L) (median, Q1 – Q3) (n=99) *** | | 0.50, 0.27-0.69 | 0.49, 0.36-0.57 | 0.75 | 0.40, 0.30-0.49 | 0.28, 0.20-0.58 | 0.28 |
| **IgG4 level (g/L) (median, Q1 – Q3) (n=99) *** | | 0.25, 0.14-0.41 | 0.70, 0.32-1.12 | 0.06 | 0.23, 0.14-0.35 | 0.26, 0.21-0.42 | 0.62 |
| **PLA_2_R1-ab level, Total-IgG ELISA U/ml (median, Q1 – Q3)** | | 1, 1-2 | 0, 0-2 | 0.22 | 139, 85-309 | 150, 97-378 | 0.52 |
| **PLA_2_R1-ab titer, Total-IgG IFT (median, Q1 – Q3)** | | 0, 0-0 | 0, 0-0 | >0.99 | 1000, 320-10000 | 2100, 320-10000 | 0.66 |
| **PLA_2_R1-ab titer, IgG4-specific IFT (median, Q1 – Q3)** | | 0, 0-0 | 0, 0-0 | >0.99 | 100, 10-100 | 100, 100-100 | 0.40 |
| **THSD7A-ab titer, Total-IgG IFT (median, Q1 – Q3)** | | 320, 100-1000 | 1000, 176-1000 | 0.67 | 0, 0-0 | 0, 0-0 | >0.99 |
| **THSD7A-ab titer, IgG4-specific IFT (median, Q1 – Q3)** | | 32, 10-100 | 32, 21-66 | 0.65 | 0, 0-0 | 0, 0-0 | >0.99 |

**Supplemental Table 3: Clinical baseline characteristics and outcome of patients with persistence of autoantibodies, compared to patients in whom autoantibodies became negative.** Q1 – Q3 – 1^st^ quartile to 3^rd^ quartile; PLA_2_R1-ab – PLA_2_R1-antibody; THSD7A-ab – THSD7A-antibody. * – for 73 of these patients sufficient amount of serum was available to perform measurement of all IgG-subclass-levels: 13 patients with THSD7A-associated MN and persistent antibodies and 7 patients in whom antibodies became negative as wells as 20 patients with PLA_2_R1-associated MN with persistent antibodies and 33 patients in whom antibodies became negative.

|  | | **THSD7A-ab during follow-up** | | **P-value** | **PLA_2_R1-ab during follow-up** | | **P-value** |
| --- | --- | --- | --- | --- | --- | --- | --- |
|  |  | **Negative** | **Persistent** |  | **Negative** | **Persistent** |  |
| **Number of patients** | | 10 | 15 |  | 40 | 20 |  |
| **Age - years (median, Q1 – Q3)** | | 66, 55-68 | 61, 45-76 | 0.98 | 54, 45-65 | 58, 45-65 | 0.67 |
| **Male gender (%)** | | 6 (60%) | 8 (53%) | >0.99 | 25 (63%) | 13 (65%) | >0.99 |
| **Proteinuria - g/24h (median, Q1 – Q3)** | | 6.9, 5.1-10.0 | 5.8, 3.6-7.2 | 0.40 | 6.8, 5.0-9.6 | 6.2, 4.4-8.5 | 0.32 |
| **Serum creatinine - mg/dl (median, Q1 – Q3)** | | 1.4, 1.0-1.9 | 1.0, 0.8-1.4 | 0.09 | 0.9, 0.8-1.3 | 1.0, 1.0-1.2 | 0.30 |
| **eGFR – CKD-EPI - mL/min/1.73 m^2^ (median, Q1 – Q3)** | | 43, 29-75 | 66, 48-96 | 0.14 | 84, 55-103 | 72, 57-93 | 0.34 |
| **Time from renal biopsy to study inclusion - months (median, Q1 – Q3)** | | 2.0, 0.4-6.5 | 0.5, 0.3-1.0 | 0.20 | 0.9, 0.5-2.0 | 0.4, 0.0-1.0 | 0.10 |
| **IgG1 level (g/L) (median, Q1 – Q3) *** | | 3.28, 2.47-3.84 | 1.97, 1.12-3.07 | 0.19 | 2.34, 1.20-3.13 | 2.29, 1.73-3.18 | 0.62 |
| **IgG2 level (g/L) (median, Q1 – Q3) *** | | 1.44, 1.08-2.15 | 1.13, 0.80-2.49 | 0.72 | 1.32, 0.92-2.12 | 1.66, 1.12-2.07 | 0.46 |
| **IgG3 level (g/L) (median, Q1 – Q3) *** | | 0.69, 0.54-0.72 | 0.42, 0.24-0.52 | 0.08 | 0.42, 0.30-0.47 | 0.36, 0.30-0.56 | 0.80 |
| **IgG4 level (g/L) (median, Q1 – Q3) *** | | 0.36, 0.26-0.95 | 0.21, 0.11-1.08 | 0.29 | 0.23, 0.14-0.51 | 0.24, 0.14-0.34 | 0.80 |
| **PLA_2_R1-ab level, Total-IgG ELISA U/ml (median, Q1 – Q3)** | | 1, 1-2 | 1, 0-2 | 0.46 | 129, 78-183 | 246, 97-409 | 0.12 |
| **PLA_2_R1-ab titer, Total-IgG IFT (median, Q1 – Q3)** | | 0, 0-0 | 0, 0-0 | >0.99 | 1000, 320-10000 | 1000, 830-3200 | 0.53 |
| **PLA_2_R1-ab titer, IgG4-specific IFT (median, Q1 – Q3)** | | 0, 0-0 | 0, 0-0 | >0.99 | 100, 10-100 | 100, 10-490 | 0.67 |
| **THSD7A-ab titer (median, Q1 – Q3)** | | 210, 33-830 | 1000, 320-1000 | 0.08 | 0, 0-0 | 0, 0-0 | >0.99 |
| **THSD7A-ab titer, IgG4-specific IFT (median, Q1 – Q3)** | | 32, 32-83 | 32, 10-100 | 0.77 | 0, 0-0 | 0, 0-0 | >0.99 |
| **Immunosuppressive treatment during follow-up (%)** | | 10 (100%) | 9 (60%) | 0.05 | 31 (78%) | 15 (75%) | >0.99 |
| **Remission of proteinuria (%)** | | 9 (90%) | 8 (53%) | 0.09 | 38 (95%) | 12 (60%) | <0.01 |
|  | **Relapse of proteinuria (% of patients who had a remission of proteinuria)** | 1 (11%) | 2 (25%) | 0.58 | 11 (29%) | 6 (50%) | 0.29 |
| **Relapse of THSD7A-ab (%)** | | 3 (30%) | na | na | 20 (50%) | na | na |
| **Doubling of serum creatinine (%)** | | 1 (10%) | 2 (13%) | >0.99 | 6 (15%) | 5 (25%) | 0.48 |

**Supplemental Table 4: Clinical baseline characteristics and outcome of patients in whom PLA_2_R1-ab relapsed, compared to patients with no relapse of PLA_2_R1-ab.**

Q1 – Q3 – 1^st^ quartile to 3^rd^ quartile; PLA_2_R1-ab – PLA_2_R1 antibody; THSD7A-ab – THSD7A antibody. * – for 33 of these patients sufficient amount of serum was available to perform measurement of all IgG-subclass-levels: 16 patients with no relapse of PLA_2_R1-ab during follow-up and 17 patients with relapse of PLA_2_R1-ab during follow-up.

|  | **No relapse of PLA_2_R1-ab during follow-up** | **Relapse of PLA_2_R1-ab during follow-up** | **P-value** |
| --- | --- | --- | --- |
| **Number of patients** | 20 | 20 |  |
| **Age - years (median,** **Q1 – Q3)** | 59, 47-70 | 54, 43-60 | 0.23 |
| **Male gender (%)** | 11 (55%) | 14 (70%) | 0.51 |
| **Proteinuria - g/24h (median, Q1 – Q3)** | 6.6, 4.9-9.8 | 6.8, 5.6-9.3 | 0.88 |
| **Serum creatinine - mg/dl (median, Q1 – Q3)** | 0.9, 0.9-1.3 | 0.9, 0.8-1.2 | 0.64 |
| **eGFR – CKD-EPI - mL/min/1.73 m^2^ (median, Q1 – Q3)** | 74, 54-90 | 89, 71-105 | 0.17 |
| **Time from renal biopsy to study inclusion - months (median, Q1 – Q3)** | 0.8, 0.5-2.3 | 1.0, 0.4-1.3 | 0.86 |
| **IgG1 level (g/L) (median, Q1 – Q3) *** | 2.38, 2.14-3.04 | 1.83, 1.13-3.19 | 0.18 |
| **IgG2 level (g/L) (median, Q1 – Q3) *** | 1.67, 1.01-2.15 | 1.12, 0.80-1.75 | 0.20 |
| **IgG3 level (g/L) (median, Q1 – Q3) *** | 0.45, 0.31-0.58 | 0.41, 0.24-0.46 | 0.19 |
| **IgG4 level (g/L) (median, Q1 – Q3) *** | 0.23, 0.15-0.52 | 0.21, 0.18-0.34 | 0.87 |
| **PLA_2_R1-ab level, Total-IgG ELISA U/ml (median, Q1 – Q3)** | 141, 101-362 | 122, 58-166 | 0.13 |
| **PLA_2_R1-ab titer, Total-IgG IFT (median, Q1 – Q3)** | 3200, 320-10000 | 1000, 1000-4900 | 0.81 |
| **PLA_2_R1-ab titer, IgG4-specific IFT (median, Q1 – Q3)** | 100, 78-100 | 100, 10-100 | 0.34 |
| **THSD7A-ab titer (median, Q1 – Q3)** | 0, 0-0 | 0, 0-0 | >0.99 |
| **THSD7A-ab titer, IgG4-specific IFT (median, Q1 – Q3)** | 0, 0-0 | 0, 0-0 | >0.99 |
| **Immunosuppressive treatment during follow-up (%)** | 11 (55%) | 20 (100%) | <0.01 |
| **Remission of proteinuria (%)** | 18 (90%) | 20 (100%) | 0.49 |
| **Relapse of proteinuria (%)** | 0 (0%) | 11 (55%) | <0.01 |
| **Relapse of PLA_2_R-ab (%)** | 0 (0%) | 20 (100%) | <0.01 |
| **Doubling of serum creatinine (%)** | 1 (5%) | 5 (25%) | 0.18 |
| **Follow-up time – months (median, Q1 – Q3)** | 53, 33-63 | 62, 57-69 | 0.03 |

**
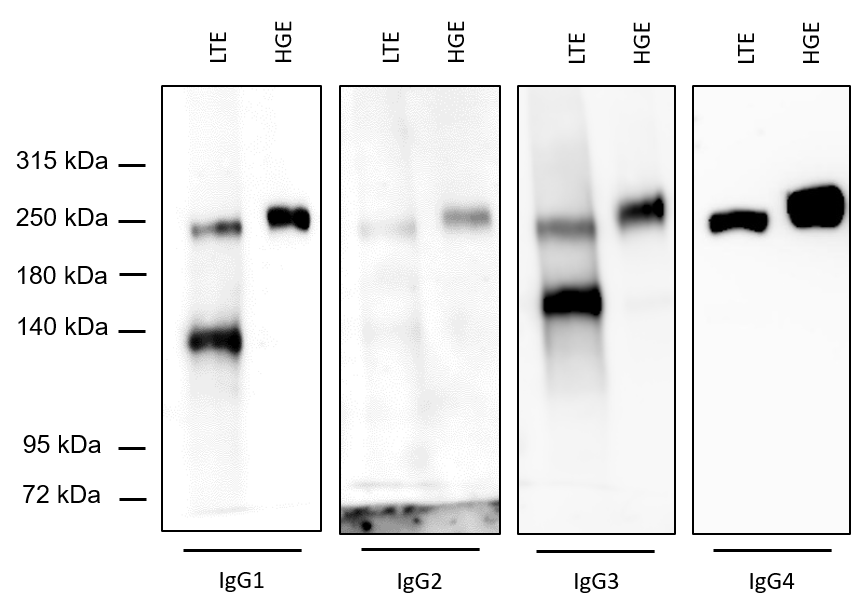
Supplemental Figure 1:** Representative Western blot analyses showing detection of THSD7A in LTE and HGE by THSD7A-ab positive human serum using the respective IgG-subclass specific secondary antibodies.

**Supplemental Figure 2**

**Negative control for the immunofluorescence staining of human kidney and lung tissue for PLA_2_R1 and THSD7A (Figure 4).**

The negative control stainings for the immunofluorescence staining of kidney (A and B) and lung (C and D) tissue were performed by omitting primary antibodies. Scale bars: 20 µm (A and C), 5 µm (B) and 10 µm (D).


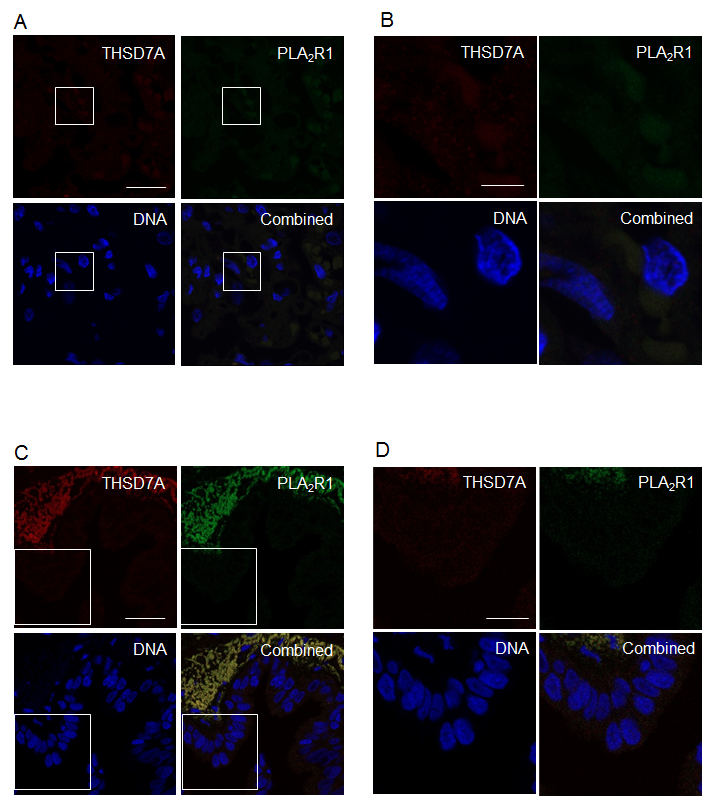

Supplement: Supplementary file 1 [file Table_1.docx]
